# Bone mineral density parameters and related nutritional factors in vegans, lacto-ovo-vegetarians, and omnivores: a cross-sectional study

**DOI:** 10.3389/fnut.2024.1390773

**Published:** 2024-06-11

**Authors:** Alexey Galchenko, Gianluca Rizzo, Elizaveta Sidorova, Elena Skliar, Luciana Baroni, Pierfrancesco Visaggi, Giada Guidi, Nicola de Bortoli

**Affiliations:** ^1^Scientific Society for Vegetarian Nutrition, Venice, Italy; ^2^Earth Philosophical Society “Melodia Vitae”, International, Toronto, CA, Canada; ^3^Independent Researcher, Messina, Italy; ^4^School of Chemical Engineering, Aalto University, Espoo, Finland; ^5^I.M. Sechenov First Moscow State Medical University (Sechenov University), Moscow, Russia; ^6^Division of Gastroenterology, Department of Translational Research and New Technologies in Medicine and Surgery, University of Pisa, Pisa, Italy; ^7^NUTRAFOOD, Interdepartmental Center for Nutraceutical Research and Nutrition for Health, University of Pisa, Pisa, Italy

**Keywords:** dual energy X-ray absorptiometry (DXA), osteoporosis, osteopenia, bone metabolism, parathyroid hormone (PTH), vitamin, trace element, acid load

## Abstract

**Introduction:**

The growing prevalence of vegetarianism determines the need for comprehensive study of the impact of these diets on health and particularly on bone metabolism. We hypothesized that significant dietary differences between vegans, lacto-ovo-vegetarians, and omnivores also cause significant differences in their nutrient status, which may affect bone health.

**Methods:**

The study assessed dual-energy X-ray absorptiometry parameters in lumbar spine and femoral neck, average nutrient intake, serum nutrient concentrations, serum PTH levels, and urinary pH among 46 vegans, 38 lacto-ovo-vegetarians, and 44 omnivores.

**Results:**

There were no differences in bone mineral density (BMD) between the groups. However, the parathyroid hormone (PTH) levels were still higher in vegans compared to omnivores, despite the same prevalence of hyperparathyroidism in all groups. These findings may probably be explained by the fact that each group had its own “strengths and weaknesses.” Thus, vegans and, to a lesser extent, lacto-ovo-vegetarians consumed much more potassium, magnesium, copper, manganese, and vitamins B_6_, B_9_, and C. At the same time, the diet of omnivores contained more protein and vitamins D and B_12_. All the subjects consumed less vitamin D than recommended. More than half of vegans and omnivores had insufficiency or even deficiency of vitamin D in the blood. Low serum concentrations of manganese with its quite adequate intake are also noteworthy: its deficiency was observed in 57% of vegans, 79% of lacto-ovo-vegetarians, and 63% of omnivores.

**Discussion:**

Currently, it is no longer possible to conclude that lacto-ovo-vegetarians have lower BMD than omnivores, as our research supported. Vegans in our study also did not demonstrate lower BMD values, only higher PTH blood concentrations, compared to omnivores, however, a large number of studies, including recent, show the opposite view. In this regard, further large-scale research is required. Vegans and lacto-ovo-vegetarians now have a variety of foods fortified with vitamins D and B_12_, as well as calcium. There is also a great diversity of ethically sourced dietary supplements. The found low concentrations of manganese require further investigation.

## 1 Introduction

More and more people exclude various products of animal origin from their diet for many reasons such as health, moral, ethical, religious, cultural, ecological and other beliefs (1–4). According to the SuperJob sociological survey (5), which involved 1,600 economically active respondents over 18 years old, in 2008, there were 3% of lacto-ovo-vegetarians in Russia. By 2013, this value had risen to 4%, but in 2021 returned to the previous value: the observed changes could be attributed to bias or related to some other reasons. Limiting the consumption of meat and offal may be, for instance, a necessary measure for cardiological and nephrological patients (6, 7). At the same time, there is an evidence (8) that this type of diets, if not properly planned, can lead to deficiency of certain nutrients (as other diets certainly can), which may contribute to metabolic disorders, including bone loss, that is, osteopenia and osteoporosis, because nutritional status has a great influence on bone metabolism.

Protein, in addition to its structural function (9), further performs a signaling role (10) by regulating the activity of osteoclast proteasomes and the balance of somatotropin and insulin-like growth factor-1 (9), which promote osteoblast activity (11) and stimulate intestinal calcium and phosphate uptake and phosphate reabsorption in the kidney (12, 13).

The inorganic component of bone is mainly represented by calcium and phosphorus (14, 15). Therefore, insufficient dietary intake of these elements can lead to demineralization of bone tissue. The composition of hydroxyapatite can also include magnesium (16–18) and zinс (19). Zinc reduces osteoclasts’ activity (20, 21) and at the same time promotes osteoblasts’ differentiation due to runt-related transcription factor 2 (RUNX2) stimulation (22). Magnesium inhibits parathyroid hormone (PTH) secretion (12) and induces osteoblasts’ proliferation (13). Moreover, copper inhibits bone resorption (23, 24) and contributes to the differentiation of stem cells into osteoblasts (23). Finally, the maturation of collagen, which is the main protein of bone tissue, occurs under the action of glycosyl and xylosyl transferases, whereas manganese serves as a cofactor for these enzymes (25).

Food has a significant impact on the acid–base state of the body. With a large acid load, reserves of alkali and alkaline earth metals are used to compensate for it, primarily calcium, the main store of which is bone tissue (26, 27). Ensuring a good supply with potassium can reduce the effect of the acid load on bone tissue (28), although the effect of acid load on calcium losses remains controversial (29). In addition, a sufficient intake of potassium reduces the body’s ability to accumulate lithium (30), an excess of which, for example, due to long-term lithium therapy for bipolar affective disorder patients, is associated with a decrease in bone mineral density (BMD) (31).

Vitamins В_6_, В_9_, and В_12_ are involved in folate cycle. Their inadequate blood levels cause the accumulation of an intermediate product, the amino acid homocysteine, in the body (32, 33). Hyperhomocysteinemia leads to a weakening of collagen bonds in bone tissue (34, 35) and a decrease in BMD can result (36). Vitamin C acts as a co-factor for prolyl and lysyl transferases engaged in collagen synthesis (37). Carotenoids reduce bone resorption (38) by inhibiting osteoclast formation (39, 40). They also constitute biological precursors of vitamin A, the alcohol form of which is retinol. The latter, in turn, stimulates the proliferation of osteoclasts (41) and can stimulate the release of lysosomal enzymes in them, eliciting bone resorption (42). Vitamin D regulates calcium and phosphate metabolism (43, 44). In addition, it stimulates osteoblast differentiation and inhibits osteoclastogenesis (45).

PTH has a significant impact on bone health. Intermittent action of this hormone enhances bone formation. However, persistently high concentrations of PTH in the blood have the opposite effect (46, 47). PTH receptors are located on both osteoblasts and osteoclasts. Activation of the former can prolong the life of osteoblasts and increase their activity. Signal transmission to the osteoclasts is slower than osteoblast stimulation. That is the reason why intermittent exposure to PTH stimulates anabolic processes (48, 49).

As a consequence, the presence of nutritional differences (status of vitamins, major and trace elements, and dietary acid load) in vegans, lacto-ovo-vegetarians, and omnivores may affect bone metabolism and increase fracture risk, as reported by other authors (50, 51).

We have not found any studies on BMD and bone health in vegetarians conducted in the Russian or any post-Soviet population, so we had a goal to represent it. The expansion of such research geography seems crucial because every region has its own climatogeographic features, and moreover, specifics of economic development, material well-being, national mentality, availability of food, and culinary preferences. Thus, the aim of our study was to crossectionally assess the state of nutritional factors potentially affecting bone metabolism in vegetarian diets (lacto-ovo and vegan), and to evaluate BMD itself in comparison to omnivorous in Russian adults.

In order to save simplicity and readability of the paper, individuals following vegan, lacto-ovo-vegetarian, or omnivorous diets will be presented as vegans, lacto-ovo-vegetarians, and omnivores, respectively. Please note that by these names authors mean nothing but dietary preferences.

## 2 Materials and methods

### 2.1 Study population

The present study included 128 adult patients: 84 vegetarians (46 vegans and 38 lacto-ovo-vegetarians) and 44 omnivorous, who were examined at the Federal Research Center for Nutrition, Biotechnology and Food Safety (Moscow) from February 2018 to December 2019. The appropriate total sample size was calculated using the G*Power software (Kiel University, Germany), with 5% error probability and 80% power (52).

We did not include to the samples subjects affected by serious somatic, neurological, and psychiatric pathologies. Since bone loss intensifies significantly with the end of reproductive age (53), especially in women (54), patients older than 50 years were also not included in the study.

Participants were divided into groups based on their own self-reported dietary pattern. The group of lacto-ovo-vegetarians included subjects who did not consume animal flesh for at least 1 year (the sample also included 3 lacto-vegetarians), and the group of vegans included subjects who did not consume any product of animal origin for at least 1 year, which corresponds to the generally accepted definitions of these dietary patterns (2).

Voluntary written informed consent was obtained from all study participants. The study was approved by the Ethics Committee of the Federal Research Center for Nutrition, Biotechnology and Food Safety (Protocol of the Ethics Committee No. 6 of December 22, 2017). This study was carried out in accordance with the World Medical Association Declaration of Helsinki (1964) and its subsequent amendments.

### 2.2 Densitometry

Dual energy X-ray absorptiometry (DXA) was chosen for the bone tissue examination. It is the gold standard for osteoporosis diagnosis, due to the relatively low radiation exposure. DXA results can be expressed as “areal bone mineral density” (g/cm^2^), which we shall henceforth call “BMD,” or as a ratio to two norms such as the BMD of the reference population by age and ethnicity (Z-score) or the BMD of the reference population of young people aged 20–30 (T-score) (55). According to the Russian clinical guidelines on diagnosis of osteoporosis, T-score should be used when working with peri and postmenopausal women and men aged over 50, who were excluded from our study (54). For this reason, we used Z-score, which is recommended for the other cases. The World Health Organization defines osteopenia when Z-score ranges from −1.0 to −2.5, while Z-score below −2.5 is a characteristic of osteoporosis (similar values are used for T-score) (56). DXA parameters such as Z-score and bone BMD were measured with a Stratos X-ray osteodensitometer (DMS, France, 2018) at the lumbar spine and at the femoral neck.

### 2.3 Laboratory analyzes

The blood samples were inoculated to Lab-Vac test tubes and then centrifuged, which allowed to separate serum from formed elements. After that, serum was transferred to micro-centrifuge tubes and frozen at a temperature of −80 degrees Celsius for up to 3 months. PTH analysis was performed immediately after the samples were obtained.

The serum concentrations of elements were determined by mass spectrometry with inductively coupled plasma (ICP-MS). For analysis, serum samples weighing about 0.8 g were placed in polypropylene centrifuge tubes and brought to a volume of 15 mL with a solution based on deionized water with a resistance of at least 15 MΩ * cm^2^ and with added 1% 1-butanol (# 1.00988, Merck KGaA, Germany), 0.1% Triton X-100 (Sigma #T9284 Sigma-Aldrich, United States), 0.07% nitric acid (Fluka #02650 TraceSELECT^™^, United States). Thus, the prepared sample was analyzed by ICP-MS on a NexION 300D quadrupole mass spectrometer (PerkinElmer, United States, s/n 81DN2062801) complete with a computer and specialized software.

Folate and vitamin B_12_ concentrations in serum were determined by chemiluminescent immunoassay using standard kits “Immulite 2000 Folic acid” and “Immulite 2000 B_12_” on Immulite 2000 analyzer (Siemens, Germany). The level of vitamin B_6_ was determined by microbiological method using the “ID-Vit^®^ Vitamin B_6_” and “Immundiagnostik AG” (Germany) kits. To determine the concentration of 25-hydroxyvitamin D in blood, chemiluminescent immunoassay was performed on ADVIA Centaur automatic analyzer with “Siemens 25-OH Vitamin D” kits (Germany). Vitamin C was determined using a Surveyor high-performance liquid chromatographic system with a PDA Plus detector and XCalibur software (Thermo, United States).

PTH level was determined using enzyme-linked immunosorbent assay (ELISA) on Immulite 2000 analyzer (Siemens, Germany).

Urine acidity was analyzed using an automated LabUMat test strip meter.

### 2.4 Dietary assessment

The nutritional assessment of the subjects was carried out using the Nutrilogic program (Nutrilogic LLC, Russia), which was developed in cooperation with the Federal Research Center for Nutrition, Biotechnology and Food Safety for nutrition specialists to evaluate the patients` dietary anamneses (57). The effectiveness of the Nutrilogic service for analyzing human nutrition and the diet’s chemical composition and its validity were confirmed in 2018 at the Moscow State University of Food Production through comparison with the results of classic calculation procedure with food tables (58).

Since we assessed the yearly nutrition of the subjects, the food-frequency method was chosen. One interview lasting 30–70 min was conducted with each subject in the format of food frequency (and amount) questionnaires. The collection of information on nutrition was carried out under the supervision of the specialist (A.G.) at the Federal Research Center for Nutrition, Biotechnology and Food Safety. The interview was fully structured. A few days before the interview, each subject was asked to recall the features of their diet and the frequency of consumption of dishes and products. During the conversation, the interviewer invited the participant to evaluate their consumption of each group of products separately: whether the subject had consumed this product during the year, how often, and in which quantity. The following intervals were used to assess the frequency of food consumption: “once a day,” “2–4 times a week,” “once a week,” “2 times a month,” “once a month,” “2–4 times a year.”

The amount of food was estimated in grams using photographic measures. For this, color images of portions of various products and dishes with a known weight from the Nutrilogic program package were used. Portions of various weights were presented in life-size photographs on a screen, and the subject determined how much their average portion was commensurate with the one depicted. The Russian national food composition database “Chemical composition and calorie content of Russian food” by V.A. Tutel’yan was used as a food composition database for the analysis (59). The handbook contains data both on raw products and cooked dishes, thus, all the technological loses were taken into account.

In the Russian Federation, food fortification is not mandatory and not widely spread overall, especially in adults, so we did not evaluate fortified food consumption. Supplements were also not considered in our study.

### 2.5 Statistical analysis

Absolute values of nutrient intake were determined for each group. The levels of PTH, chemical elements, and vitamins in the blood, and the values of pH and DXA parameters were evaluated. The results obtained by the dietary assessment were compared with the daily nutritional requirements set in the Russian Federation (60, 61). The blood levels of chemical elements were compared with the normative values calculated by the laboratory “Micronutrients LLC” (Moscow), the other blood tests` reference values were taken as set by the Federal Research Center for Nutrition, Biotechnology, and Food Safety (Moscow). In each group, the percentage of people who consumed an inadequate amount of nutrients, with an inappropriate concentration of PTH or abnormal levels of nutrients in the serum was calculated. Densitometry criteria were compared with reference values for osteopenia and osteoporosis, and the proportion of people with these conditions was calculated.

The normality of continuous variables was checked by Shapiro–Wilk test. If normal, a variable was shown as mean (SD) and the t-test for independent samples was used to compare 2 groups. If not normal, a variable was presented as median (interquartile range) and the Mann–Whitney test was applied to compare 2 groups. Bonferroni post-hoc correction was applied to reduce the risk of false-positive results in multiple comparisons. Correlations were estimated by Pearson’s coefficient or Spearman rank correlation tests, where appropriate. The comparison of proportions was performed by Pearson’s χ^2^ test, Pearson’s χ^2^ test with Yates’s continuity correction, or Fisher’s exact test. *p*-value less than 0.05 was considered as statistically significant. All analyses were performed with SPSS 23 (IBM, United States).

## 3 Results

There were no significant differences in age and BMI between the groups. Anthropometric characteristics of the groups are shown in Table 1.

**Table 1:** Demographic and anthropometric characteristics of the vegan, lacto-ovo-vegetarian and omnivore participants.^1^

	*n* = 128		Age	BMI
	Male	Female	ME (25%; 75% percentiles)	ME (25%; 75% percentiles)
Vegans	22	24	31 (29; 33)	21 (20; 23)
Lacto-ovo-vegetarians	12	26	34 (27; 36)	21 (19; 23)
Omnivores	16	28	33 (29; 38)	24 (23; 25)

^1^for all, *p* > 0.05.

### 3.1 Densitometry

No significant differences in mean DXA parameters were found among the dietary groups. Twenty-six percent of vegans were diagnosed with osteopenia following the lumbar spine investigation. The same condition was detected in 19% of vegans, when examining the femoral neck. In 43% of lacto-ovo-vegetarians, the level of mineralization at the lumbar spine corresponded to osteopenia. The left femoral neck DXA showed a similar result in 20% of lacto-ovo-vegetarians. Among omnivores, osteopenia was found in 32% at the lumbar spine and in 13% at the left femur. Also, only among lacto-ovo-vegetarians, it was observed 4% of subjects with osteoporosis at the femoral neck. However, there were no statistically significant differences between the groups (Table 2).

**Table 2:** DXA parameters among vegan, lacto-ovo-vegetarian, and omnivore groups measured at the left femoral neck and at the lumbar spine.^1^

	DXA parameters	Vegans	Lacto-ovo-vegetarians	Omnivores
		M ± SD	M ± SD	M ± SD
Lumbar spine	Z-criterion	0.000 ± 1.334	−0.465 ± 1.367	0.200 ± 1.467
	BMD^2^, g/cm^2^	1.074 ± 0.157	1.071 ± 0.176	1.072 ± 0.187
Hip neck	Z-criterion	0.183 ± 1.063	−0.088 ± 1.081	0.183 ± 0.754
	BMD^2^, g/cm^2^	1.042 ± 0.170	0.986 ± 0.175	1.052 ± 0.132

^1^for all, *p* > 0.05.

### 3.2 PTH

The PTH levels in vegans were significantly higher than in omnivores (*p* < 0.001), although the mean (median) values fell within the normal range. No other significant differences between the groups were observed. The prevalence of hyperparathyroidism also did not differ (Table 3).

**Table 3:** PTH blood levels and prevalence of hyperparathyroidism in vegans, lacto-ovo-vegetarians, and omnivores.

	Vegans ME (25%; 75% percentiles)	Lacto-ovo-vegetarians ME (25%; 75% percentiles)	Omnivores ME (25%; 75% percentiles)	Normal PTH range
PTH blood levels, pg/ml	47.2 (42.1; 59.5)^1,2^	37.6 (35.0; 49.8)^2^	39.2 (37.6; 41.3)	11–67
Prevalence of hyperparathyroidism	8%	5%	5%	-

^1^vs. omnivores, *p* < 0.001. ^2^normal distribution (M ± SD): vegans 50.6 ± 20.0; lacto-ovo-vegetarians 43.2 ± 16.2.

### 3.3 Nutritional intake

Omnivores consumed significantly more protein: a mean of 70 g/day, compared to 54 g/day among lacto-ovo-vegetarians and 58 g/day among vegans. While calcium intake did not differ significantly between the groups (Table 4), the percentage of people intaking calcium less than the recommended 1,000 mg per day was higher among omnivores than among lacto-ovo-vegetarians (Table 5).

**Table 4:** Intake of chemical elements and vitamins among vegans, lacto-ovo-vegetarians, and omnivores.

	Vegans	Lacto-ovo-vegetarians	Omnivores	Recommended consumption in Russia (61)	Nutrient recommendations: dietary reference intakes (DRI) (62)
	ME (25%; 75% percentiles)	ME (25%; 75% percentiles)	ME (25%; 75% percentiles)		
K, mg/day	7,071 (5,362; 8,693)^2,3,5,6^	3,732 (3,245; 4,652)^7^*	2,622 (2,371; 3,247)	3,500	2,600 (♀ age 19 and older), 3,400 (♂ age 19 and older)
Mg, mg/day	674 (479; 934)^2,3,5,6^	401 (291; 531)^7^	288 (264; 331)	420	310 (19–30 ♀), 320 (31–50 ♀), 400 (31–50 ♂), 420 (31–50 ♂)
Ca, mg/day	787 (582; 1,056)	852 (630; 988)	738 (628; 822)	1,000	1,000 (19–50 and ♀19–70 years ♂)
P, mg/day	1,125 (884; 1,461)	1,077 (707; 1,386)*	1,097 (954; 1,237)	700	700 (age 19 and older)
Zn, mg/day	7.6 (5.7; 9.1)^2,3^	5.8 (3.5; 7.9)^8,9^*	7.6 (6.7; 8.0)	12.0	8.0 (♀ age 19 and older), 11.0 (♂ age 19 and older)
Cu, mg/day	2.4 (1.9; 3.0)^2,3,5,6^	1.7 (1.1; 2.1)^7^*	1.1 (1.0; 1.2)	1.0	0.9 (age 19 and older)
Mn, mg/day	6.4 (4.6; 8.2)^1,5^	5.1 (3.0; 7.3)^7^	4.6 (3.7; 5.8)	2.0	1.8 (♀ age 19 and older), 2.3 (♂ age 19 and older)
D, μg/day	0.00 (0.00; 0.00)^2,3,5,6^	0.29 (0.07; 0.75)^8,9^	0.99 (0.57; 1.14)	15.0	15.0 (19–70 years)
B_6_, mg/day	3.4 (2.6; 3.9)^2,3,5,6^	1.7 (1.3; 2.0)^7^	1.4 (1.2; 1.6)	2.0	1.3 (19–50 years)
B_9_, μg/day	508 (400; 553)^2,3,5,6^	333 (249; 429)^7,9^	252 (197; 280)	400	400 (age 19 and older)
B_12_, μg/day	0.00 (0.00; 0.00)^1,4,6^	0.29 (0.03; 0.51)^7,9^	2.34 (1.97; 3.49)	3.0	2.4 (age 19 and older)
C, mg/day	430 (325; 575)^1,3,4,6^	206 (156; 277)^8^	128 (88; 140)	100	75 (♀ age 19 and older), 90 (♂ age 19 and older)
RE, μg/day	1,138 (756; 1,592)^2,3,5,6^	767 (610; 847)	719 (555; 961)	900	700 (♀age 19 and older), 900 (♂ age 19 and older)

^1^vs. lacto-ovo-vegetarians, *p* < 0.05; ^2^vs. lacto-ovo-vegetarians, *p* < 0.001; ^3^vs. lacto-ovo-vegetarians after Bonferroni correction, *p* < 0.05; ^4^vs. omnivores, *p* < 0.05; ^5^vs. omnivores, *p* < 0.001; ^6^vs. omnivores after Bonferroni correction, *p* < 0.05; ^7^vs. omnivores, *p* < 0.05; ^8^vs. omnivores *p* < 0.001; ^9^vs. omnivores after Bonferroni correction, *p* < 0.05; *normal distribution (M ± SD): K 3891.9 ± 1477.4; *P* 1049.9 ± 386.0; Zn 5.8 ± 2.6; Cu 1.6 ± 0.7; RE: retinol equivalent.

**Table 5:** Prevalence of chemical elements and vitamins intake insufficiency among vegans, lacto-ovo-vegetarians, and omnivores.

	Vegans % (n)	Lacto-ovo-vegetarians % (n)	Omnivores % (n)	Recommended consumption in Russia (61)
K	7% (3)^1,4^	29% (11)^5^	77% (34)	3,500 mg/day
Mg	13% (6)^2,3^	66% (25)^6^	91% (40)	420 mg/day
Ca	65% (30)	74% (28)^5^	89% (39)	1,000 mg/day
P	9% (4)	21% (8)	7% (3)	700 mg/day
Zn	83% (38)	97% (37)	82% (36)	12.0 mg/day
Cu	4% (2)^4^	21% (8)	34% (15)	1.0 mg/day
Mn	0% (0)	0% (0)	0% (0)	2.0 mg/day
D	100% (46)	100% (38)	100% (44)	15.0 μg/day
B_6_	17% (8)^2,4^	68% (26)^5^	86% (38)	2.0 mg/day
B_9_	24% (11)^2,4^	71% (27)^5^	88% (39)	400 μg/day
B_12_	100% (46)^4^	100% (38)^6^	70% (31)	3.0 μg/day
C	0% (0)^1,3^	16% (6)	25% (11)	100 mg/day
RE	30% (14)^1,4^	76% (29)	73% (32)	900 μg/day

^1^vs. lacto-ovo-vegetarians, *p* < 0.05; ^2^vs. lacto-ovo-vegetarians, *p* < 0.001; ^3^vs. omnivores, *p* < 0.05; ^4^vs. omnivores, *p* < 0.001; ^5^vs. omnivores, *p* < 0.05; ^6^vs. omnivores *p* < 0.001; RE, retinol equivalent.

Vegans consumed significantly more potassium, magnesium, and copper than lacto-ovo-vegetarians and omnivores and had a lower risk of deficiency of these elements in their diet. Lacto-ovo-vegetarians occupied an intermediate position (whether any significant differences were observed) in terms of intake of all chemical elements except zinc: they consumed it less than other groups, but there were no significant differences among the groups in the occurrence of zinc intake below the recommended level (Tables 4, 5).

The consumption of almost all vitamins, except D and B_12_, increased with the decrease in the proportion of animal-derived food in the diet. Vitamins D and B_12_ were maximally represented in the diet of omnivores (Table 4). However, the risk of insufficient intake of these vitamins was present in the overwhelming number of the subjects from all groups (Table 5).

### 3.4 Serum nutrient levels

Despite the higher amount of copper in the diet of vegans, its concentrations in the blood were, on the contrary, lower than in omnivores, although after using Bonferroni correction, the differences were not supported (Table 6).

**Table 6:** Serum levels of chemical elements and vitamins among vegans, lacto-ovo-vegetarians, and omnivores.

	Vegans	Lacto-ovo-vegetarians	Omnivores
	ME (25%; 75% percentiles)	ME (25%; 75% percentiles)	ME (25%; 75% percentiles)
K, μg/ml	246 (201; 259)	243 (225; 252)	240 (201; 252)
Mg, μg/ml	22.3 (21.8; 23.0)^4^	22.5 (21.9; 24.1)	23.5 (21.6; 24.4)
Ca, μg/ml	98.6 (94.5; 102.0)^1,4^	106.0 (101.0; 109.0)	103.5 (98.0; 108.0)*
Zn, μg/ml	0.94 (0.87;1.02)^5,6^	0.99 (0.85; 1.12)^8^*	1.10 (1.05; 1.22)
Cu, μg/ml	1.00 (0.87; 1.18)^4^	1.09 (1.02; 1.15)	1.24 (1.08; 1.39)
Mn, μg/ml	0.0013 (0.0011; 0.0018)	0.0013 (0.0012; 0.0015)	0.0014 (0.0011; 0.0016)
D, ng/ ml	21.8 (14.9; 29.3)	15.1 (11.4; 23.4)	22.9 (18.1; 29.3)
В_6_, nmol/ml	19.7 (17.0; 24.9)^2,5,3,6^	21.7 (17.9; 28.1)^8^	15.9 (14.6; 19.4)
В_9_, nmol/ml	9.11 (7.55; 14.10)^1,4^	7.62 (5.95; 9.77)*	5.87 (4.68; 9.50)
В_12_, pg/ml	193 (150; 309)^1,4^	290 (202; 364)^7^	323 (278; 522)
С, μg/ml	19.8 (16.7; 22.9)	21.7 (17.1; 26.5)*	18.6 (17.1; 24.0)

^1^vs. lacto-ovo-vegetarians, *p* < 0.05; ^2^vs. lacto-ovo-vegetarians, *p* < 0.001; ^3^vs. lacto-ovo-vegetarians after Bonferroni correction, *p* < 0.05; ^4^vs. omnivores, *p* < 0.05; ^5^vs. omnivores, *p* < 0.001; ^6^vs. omnivores after Bonferroni correction, *p* < 0.05; ^7^vs. omnivores, *p* < 0.05; ^8^vs. omnivores *p* < 0.001; ^9^vs. omnivores after Bonferroni correction, *p* < 0.05; *normal distribution (M ± SD): Ca 101.9 ± 9.8; Zn 0.9 ± 0.2; B_9_ 8.2 ± 2.5; C 21.7 ± 4.5.

In general, after correction, only zinc levels in the blood of omnivores remained significantly higher compared to vegans (Table 6). The risk of deficiency of major and trace elements in the serum did not differ between the dietary groups (Table 7). Deficiency of any element in the blood, except manganese, was observed in no more than 11% of vegans, 5% of lacto-ovo-vegetarians, and 5% of omnivores. Manganese deficiency was extremely widespread. It was found in more than half of subjects from all groups.

**Table 7:** Prevalence of chemical elements and vitamins deficiency in the blood of vegans, lacto-ovo-vegetarians and omnivores.

	Vegans % (n)	Lacto-ovo-vegetarians % (n)	Omnivores % (n)
K	0% (0)	0% (0)	0% (0)
Mg	0% (0)	0% (0)	0% (0)
Са	0% (0)	0% (0)	2% (1)
Zn	0% (0)	3% (1)	0% (0)
Cu	11% (5)	5% (2)	5% (2)
Mn	57% (27)	79% (30)	63% (28)
D, 20–30, ng/ml	34% (16)^1^	12% (5)^3^	38% (17)
D, <20, ng/ml	41% (19)^1^	18% (7)^3^	38% (17)
В_6_	2% (1)	5% (2)	9% (4)
В_9_	0% (0)^1,2^	16% (6)^3^	65% (27)
В_12_	28% (13)^1,2^	8% (3)	0% (0)
C	0% (0)	0% (0)	0% (0)

^1^vs. lacto-ovo-vegetarians, *p* < 0.05; ^2^vs. omnivores, *p* < 0.001; ^3^vs. omnivores, *p* < 0.05.

Serum vitamin D concentrations did not differ among the subjects (Table 6), and most were below the recommended level. According to the recommendations of the Union of Pediatricians of Russia (63), insufficient vitamin D level is defined with a calcidiol level from 20 to 30 ng/mL. Vitamin D deficiency is defined if 25(ОН)D concentration falls below 20 ng/mL. Interestingly, despite the absence of significant differences in serum concentrations, lacto-ovo-vegetarians had the lowest prevalence of both vitamin D insufficiency and deficiency in the blood (Table 7). The latter could be due to their consumption of milk fortified with vitamin D, although, as already mentioned, such products are not widely spread in the region of study.

The level of vitamin B_9_ in the serum increased with an increase in the proportion of plant foods in the diet. The reverse trend was observed for vitamin B_12_. Vitamin B_6_ concentrations were highest in lacto-ovo-vegetarians, but the frequency of deficiencies remained similar to the vitamin B_9_ trend (Tables 6, 7).

### 3.5 Urinalysis

The urine pH levels were significantly lower in omnivores than those in vegans and lacto-ovo-vegetarians were (*p* = 0.000 and *p* = 0.02, respectively) and amounted to 5.6 (Me; 4.8 (25th percentile); 6.2 (75th percentile)). pH values among vegans and lacto-ovo-vegetarians were 6.4 (6.0, 7.4) and 6.3 (5.8, 6.9), respectively. There were no significant differences in the results of the analysis between vegans and lacto-ovo-vegetarians.

### 3.6 Correlation analysis

Neither consumption nor serum levels of the assessed nutrients were associated with the BMD or PTH levels. It might be explained by the complexity of the multifactorial influence of nutrition on BMD: a good supply with some important for bone health nutrients was accompanied by a poor supply with the other ones.

## 4 Discussion

### 4.1 BMD

Although no significant differences in DXA indicators were found among our dietary groups, it is worth noting that Z-criteria in lacto-ovo-vegetarians were slightly lower than in the other groups, and the percentage of people with osteopenia was higher. Lacto-ovo-vegetarians were also the only group where osteoporosis was found. However, lacto-ovo-vegetarians, although not statistically significant, were still somewhat older than their counterparts.

Research on bone health in vegetarian and plant-based diets is overall not consistent. In a 2009 meta-analysis, the BMD of vegetarians (lacto-ovo-vegetarians and vegans) was lower than that of omnivores (64), which was further found in 2018 meta-analysis (65). Movassagh et al. even showed with DXA that the BMD of lacto-ovo-vegetarians may be higher than that of omnivores (66) and, moreover, that lacto-ovo-vegetarian diet has long-term positive impact on BMD. The opposite view was presented in a 2022 study by Chuang et al. (67), in which they described larger reduction in lumbar spine BMD for 3 years in vegetarian (lacto-ovo and vegan) woman, compared with non-vegetarians, aged 40–55.

In a 2019 study by Xie et al., BMD of lacto-ovo-vegetarians and omnivores, measured via ultrasound densitometer, did not differ (68), yet the BMD of vegans was still significantly lower. In a 2021 cross-sectional study, vegans also had lower ultrasound BMD parameters than omnivores (69). It seems therefore, that some components of animal origin, which are characteristic of lacto-ovo-vegetarian diet, may play a special role in BMD formation. Thus, positive association was observed between whole-body T-score and egg consumption (70). A 2021 meta-analysis (71) provided that dairy products also can increase BMD, however, in the latest study (72) dairy food intake was not associated with bone texture.

In our study, the BMD of lacto-ovo-vegetarians did not significantly differ from that of omnivores, which is consistent with results of some recent studies. The BMD of vegans also was not significantly lower, in contrast to most other results. In any case, great assortment of participants’ ages in every single study makes it difficult to compare the outcomes. They are difficult to compare not only because participants of different ages have, as expected, different BMDs. It is known that bone mass gain occurs until the age of 30 years (predicted medians are 22.31 (21.95; 22.59) for females and 26.86 (25.14; 27.98) for males) (73), and after this age, its decrease begins. Accordingly, studies involving participants under 20 years of age evaluate the effect of diet or lifestyle primarily on the processes of osteosynthesis, while studies with an age group of 30 years and older assess the effect of vegetarianism (lacto-ovo and/or veganism) primarily on the processes of bone resorption. Moreover, the improvement in the quality of vegetarian diets, due to an increase of the knowledge in recent times of the correct planning of such diets and the role of nutrients in human health (8) may be responsible of better outcomes on previous pitfalls.

### 4.2 PTH

In most studies, PTH levels among lacto-ovo-vegetarians and vegans were significantly higher than those of omnivores (74–76). We observed significantly higher concentrations of PTH in vegans in contrast to omnivores, though prevalence of hyperparathyroidism did not differ between the groups. This is most likely due to the fact that they consume significantly less vitamin D and have a higher risk of its deficiency (77). However, some authors found the opposite picture. In their studies, PTH blood concentrations were lower in the people with plant-based diets (78, 79).

### 4.3 Nutritional status

Low protein intake usually increases the risk of fractures (80–82). On the other hand, the Nurse’s Health Study found no association between protein consumption and fracture risk (83). Moreover, some studies have revealed that people with a high proportion of protein in their diet had a higher rate of bone resorption (84). Lacto-ovo-vegetarian and vegan diets usually satisfy the body’s need for protein and all 20 amino acids (85–87). At the same time, vegans still need to pay attention to protein and, especially, sulfur-containing amino acids consumption, since even with an adequate amount of total protein intake, they may sometimes be deficient in methionine and cysteine due to their lower content in some staple protein-rich plant foods (88).

In our study, the amounts of protein in the diet of lacto-ovo-vegetarians and vegans were at the same level, lower than those of omnivores. The same has been found in other studies (89–93). The recommended daily protein intake according to the Russian prescriptions equals to 75–114 g/day for men and 60–90 g/day for women (according to the US RDA, 0.8 g/kg of body weight per day for both sexes). However, individual protein requirements are highly variable and depend on plenty of factors that were not estimated in this study (94), Thus, we decided not to use these reference values to compare inadequate protein intake between the groups. We should also note, that, naturally, the recommended intake levels usually mean the amount of protein, as well as any other nutrient, constant daily consumption of which with food suffices to prevent the development of its deficit in 97.5% of the almost healthy population, given its bioavailability (95). Accordingly, average nutrient requirements are significantly lower than the recommended daily intakes.

Insufficient calcium intake can lead to bone demineralization and a decreased BMD (8, 96, 97). As a recent meta-analysis (98) demonstrates, vegan diets usually contain less calcium, which may increase the risk of osteoporosis (99), although in 2021 the study by Jakše et al. showed the reverse picture (100). Additionally, 2020 review concluded that plant-based diets with adequate calcium and vitamin D levels do not have any detrimental effects on bone health (101). In principle, lower-than-optimal calcium intake is common in most diets. In our study, there were no significant differences in calcium intake among dietary groups, although its levels in the blood were significantly lower in vegans. Similar serum test results were found in other studies (69, 76, 91, 102, 103). However, circulating calcium concentration cannot be considered as the best indicator of calcium status in the body, since its blood levels, as other electrolytes’ (including potassium and magnesium), vary not much because of strict homeostatic control (104). The bioavailability of calcium is reduced in many products due to the presence of phytic and oxalic acids that inhibit its absorption (14). Low content of these acids in some green leafy vegetables that contain low oxalate (such as kale, Brussel sprouts, broccoli) and tofu makes them a good source of calcium for vegans, while for lacto-ovo-vegetarians it is also dairy products (1, 105–110). Mineral waters may be considered as another good source of calcium for any dietary group (111).

Phosphorus in food products is distributed very widely and evenly (105), therefore, its deficiency is rare among all dietary groups (90, 91, 103, 112–115). In addition, phosphorus intake is quite difficult to estimate today, since phosphate supplements are widespread (44).

Potassium and magnesium are usually found in adequate quantities in plant-based diets. Often, a decrease in animal-derived food consumption rises potassium and magnesium intake (114, 116–119). Among omnivores, magnesium deficiency in Western societies is common (8, 120–122).

A number of studies support the higher intake of copper by lacto-ovo-vegetarians and vegans (91, 123). It is important to note that copper absorption occurs mainly in the stomach, and dietary phytates do not interfere with this process (124). However, the bioavailability of copper in plant-based diets is still somewhat lower than in omnivorous diets due to the reduction potential of larger quantities of vitamin C (125). This explains the lower concentration of copper in the blood of vegans compared to omnivores.

In a plant-based diet, manganese deficiency is unusual (124, 126, 127), and in our study high enough intake was observed. Surprisingly, at the same time, its deficiency was widely distributed. Since manganese is an essential trace element, its status in different populations deserves further studies.

Low intake of zinc is widespread. In our study, zinc consumption was the lowest among lacto-ovo-vegetarians, as well as its serum levels among subjects who followed plant-based diets were, because the absorption of this element is inhibited by oxalic and phytic acids (1, 115, 128, 129). Despite the fact that activity of phytates decreases during cooking (130, 131), recommended values of zinc intake by vegetarians (lacto-ovo and vegans) are about 1.5 times higher (14, 132).

30% of all the subjects in our study had vitamin D insufficiency (20–30 ng/mL) and 34% had deficiency (<20 ng/mL) of this nutrient. 100% of the participants consumed insufficient quantities of Vitamin D. Many studies support that with an increase in the proportion of products of animal origin, the intake of this vitamin increases (91, 103, 114, 116, 119, 123). The level of vitamin D in the serum of omnivores has been reported higher compared to vegans (53, 76, 133). However, we observed almost identical calcidiol concentrations in vegans and omnivores. Similar results were obtained in some other studies (80, 134). Most of the subjects in all studies had vitamin D deficiency, which indicates the mass nature of this problem, regardless of the type of diet. Moreover, the prevalence of vitamin D deficiency increases among countries without mandatory fortification, which is one of the main determinants of the vitamin status (135) and with increasing latitude, as the cutaneal synthesis of calciferol becomes impossible in areas with low insolation (136). It seems therefore that vitamin D status relies predominantly on the lifestyle and insolation regime, but not on the type of diet (77). Thus, in the Vietnamese study (53), vitamin D levels among all subjects were slightly higher than in other investigations, while as our recent study shows, even mild supplementation is insufficient for vegans in Russia in most cases (137).

Lacto-ovo-vegetarians and vegans usually consume adequate (113, 123) and often higher (138) amounts of vitamin В_6_ than omnivores do. Serum concentrations of this vitamin are often similar in people with different eating behaviors (103, 134). Vegans had the highest intake of vitamin B_6_ in our study. Folate intake in plant-based diets (89–91, 103, 112, 119, 123, 139) and the blood levels of this vitamin in vegetarians (lacto-ovo and vegans) (103, 126, 140) are usually higher than in omnivorous ones, which corresponds to our results. In many studies, the intake of vitamin B_12_ in all dietary groups was noticeably higher than it was in our study (91, 103, 116, 123). Cobalamin consumption is limited in a diet including few animal-derived products or excluding them, since plants do not accumulate (or synthesize) it. As a result, vegetarians (lacto-ovo and, especially, vegans) are more predisposed to lower concentrations of vitamin B_12_ in the serum if not supplement it (103).

Vitamin C intake in all groups in our study often exceeded other results (103, 114, 116, 117, 133). In general, among lacto-ovo-vegetarians and vegans, vitamin C consumption is higher than among omnivores. This also reflects the level of this vitamin in the blood (103, 134).

Vegetarians (lacto-ovo and vegans) consume a bit of retinol (118, 123, 140), unlike omnivores do (141), but the large amount of carotenoids ingested by them provides high total values of retinol equivalent (RE) (118, 140). As for retinol concentrations, there are usually no significant differences between the groups (103, 134, 142).

### 4.4 Acid load

Barzel et al. believe that animal protein significantly enlarges acid load, which increases bone resorption (143). The negative effect of acidity on bone tissue is explained by the fact that extracellular H^+^ stimulate activity of osteoclasts (144), which in turn produce hydrogen ions for bone resorption (145). The pH of the urine that we measured might have reflected the pH of the blood, since when the acidity of the serum increases, excess protons are excreted by the kidneys (146). Thus, the higher acidity of urine we found in omnivores may have indicated the higher acid load of serum in their diet.

In Western countries, typical omnivorous diets mainly reduce blood pH by about 50–70 mEq/day (53). Plant-based diets, on the contrary, have an alkalizing effect (117, 147–149). The effect of different diets on serum pH was compared by Knurick et al.: +19.6 mEq/day for omnivores, −1.5 mEq/day for lacto-ovo-vegetarians, and − 15.2 mEq/day for vegans (89). A significant alkalizing effect of food was also found in vegans in Germany (−39 mEq/day) (117). The vegan diet, which has the most pronounced alkalizing effect, could slow down the resorption of bone tissue and promote its synthesis. In general, sufficient consumption of vegetables has a beneficial effect on bone tissue (147).

Nevertheless, a meta-analysis by Fenton et al. found no evidence that increasing the diet acid load promotes skeletal bone mineral loss or osteoporosis (29).

### 4.5 Strengths and limitations

Our study has several strengths: the first one relies on having separately examined vegetarians as lacto-ovo-vegetarians and vegans, since we could dispose of dietary samples of comparable size. The second strength is that a large array of the most important parameters linked to bone metabolism has been evaluated. Another strength is the geography of the study. All the previous studies on this topic have been conducted in Western Europe, North America or South-East Asia. Thus, we spread the study geography quite far from those 3 parts of world.

Some limitations are also present. No statistically significant difference in BMD was detected in the different dietary groups, but the results have not been adjusted for other confounding variables, which were not evaluated, such as lifestyle habits, physical activity, use of supplements, state of microbiota (12). These factors can vary between groups and elicit a significant impact on the research outcomes, as was mentioned earlier (150). The cross-sectional design of the study could not provide a causal effect of diet on BMD and osteoporosis. To have any chance to speculate about the cause-effect relationships, a longitudinal study is required, and its duration probably should last for decades. As BMD changes gradually over the course of life, another crucial aspect for dietary effects assessment is the study design that contemplates inclusion of participants who have been following their diets for many years. In our study the minimum acceptable period of following the diet was quite short (1 year). The effectiveness of statistical data processing decreased as a result of the fact that the groups were rather small and were not divided by sex or age. Besides, although there were no differences in the age composition between the groups, the age variation was rather wide.

Another important limitation is the method we used to estimate BMD. The most accurate method for osteoporosis diagnosis is quantitative computed tomography (QCT), as it evaluates the true BMD by showing the three-dimensional genuine BMD measured in g/cm^3^ and allows to assess the spongy bone condition, with changes in which osteoporosis begins (151). However, QCT could not be used in our study because it is associated with a high radiation exposure. Thus, DXA was used as the gold standard for primary diagnosis of osteoporosis and particularly because of its low cost. In this regard, it is important to mention that DXA can give false-positive results since it is sometimes impossible to distinguish between a progressive pathological process and congenital features of bone tissue development. For instance, the cortical bone (which DXA assesses) is often rather thin in asthenic people.

Furthermore, single bone scanning is not an indicator of progressive destructive process. The osteoporosis diagnosed in our study is nothing more than the correspondence of the measured BMD value to the accepted indicator, while osteoporosis is a pathological process accompanied by a gradual decrease in the bone’s mineral composition. The disease may be present in a person with BMD within the normal range, if the process has not yet affected the cortical bone but originated in the spongy one. The exact diagnosis can be made only by the results of several studies showing whether the BMD is decreasing at the expected rate. Nevertheless, the quantification of BMD by DXA is mainly used in research studies.

Our purpose was to evaluate nutrition of the subjects throughout the year. Since we had limited resources, our choice fell on the food-frequency method, which, undoubtedly, has a big margin of error, especially, when considering a one-year period of data collection. However, supervision of the specialist during the interview significantly increased the accuracy of the study. Although 24-h dietary recall or, moreover, prospective methods are much more accurate, their using for such a long period requires considerable resources and very high participants’ motivation. At the same time, validity of the food-frequency method was shown in a number of studies (152–154). Used by us food-frequency method is not very exact, but its bias affects all the groups in the same way, so presented differences may be considered as informative enough.

## 5 Conclusion

In our study, we found that vegetarians (lacto-ovo and vegans) did not have lower BMD than omnivores. Meanwhile, higher PTH levels were observed in vegans compared to omnivores, though prevalence of hyperparathyroidism was the same in all groups. Despite vegans are reported at an increased risk of bone loss, our study did not show it.

Omnivores were better provided with vitamins D and B_12_, vegans – with all other assessed vitamins. The RE consumption values in the vegan group were significantly higher than in others. Vegans` RE intake is predominantly made up of carotenoids consumption, which have a protective effect on bones. Instead, in omnivores retinol, able to intensify bone resorption, is the main contributor to the RE intake. Vegans and lacto-ovo-vegetarians consumed more potassium, magnesium, and copper and had a slightly higher urine pH, which, in general, prevented bone resorption. The risk of deficiency of major and trace elements in the serum did not differ between the dietary groups. Conversely, omnivores had more protein in their diets compared to plant-based diets. Lacto-ovo-vegetarians occupied an intermediate position in almost all cases, where significant differences were found. Results of the present study are in line with the most recent studies on this topic (8).

Bone metabolism is affected by many nutrients, and each dietary group could have its own strengths and weaknesses in this regard. Any dietary group of temperate climates is at extremely high risk for vitamin D deficiency, and latitude and supplementation have a much greater effect on vitamin D status than dietary type. Supplementing vitamin D is necessary for every people living in an extratropical region. All types of vegetarians ought to supplement vitamin B_12_, as well [as suggested in the VegPlate (155)]. Very poor calcium intake in all dietary groups directs our attention to products with high calcium and low oxalate content (Brassica vegetables, tofu, mineral waters) in the role of primary calcium sources, as well as raises a question of calcium mandatory supplementation and spread of fortification of products with it. Moreover, omnivores need to pay an extra attention to other micronutrients: insufficient intake of potassium, magnesium, and folate is rampant in developed countries. Zinc levels are recommended to be monitored by all groups, but especially by vegans and lacto-ovo-vegetarians. Manganese intake was high in all groups, although its deficiency was frequently observed. Thus, its status requires special study.

In light of the above, it makes sense for people from the high-risk groups (for instance, people over 50 years old, especially women) to conduct DXA with a certain frequency, depending on the results of the study. In a case of low DXA indicators, it is recommended to undergo QCT scanning (151). After osteoporosis has been diagnosed, it is also desirable to examine the peripheral bones, primarily the rays, since they are at high risk of low-traumatic fractures, as well. The assessment can be done with peripheral QCT, which irradiates a relatively small volume of tissues. Screening of peripheral bones condition may also include quantitative ultrasonography. This method is only applicable for evaluating of superficially located bones and does not have a high diagnostic value. However, quantitative ultrasonography uses non-ionizing radiation, it is quite cheap and often transportable, which makes it convenient for periodic assessment of the bones condition. Besides, bone metabolism can be indirectly tracked by studying the concentrations of bone tissue metabolites: ostase, osteocalcin, procollagen type 1 N-terminal propeptide, or C- and N-terminal telopeptides of type 1 collagen in serum, as well as pyridinoline and deoxypyridinoline in urine (156).

A regular physical activity is the basis of recommendations for the prevention of BMD reduction (12, 157). R. Wakolbinger-Habel et al. in their study found that BMD in non-exercising vegans was lower when compared with a similar group of people with an omnivore diet, although there were no differences in the microarchitecture of bone tissue between vegans and omnivores who had regular strength training (158).

Future studies should contain a comprehensive assessment of nutritional status with a larger number of subjects, divided into dietary, sex and age groups. In addition, further expansion of the geography of such studies is needed due to significant regional features of the chemical composition of soils, religious, cultural, culinary traditions, and economic status.

## Data availability statement

The raw data supporting the conclusions of this article will be made available by the authors, without undue reservation.

## Ethics statement

The studies involving humans were approved by Ethics Committee of the Federal Research Center for Nutrition, Biotechnology and Food Safety. The studies were conducted in accordance with the local legislation and institutional requirements. The participants provided their written informed consent to participate in this study.

## Author contributions

AG: Conceptualization, Data curation, Formal analysis, Funding acquisition, Investigation, Methodology, Project administration, Resources, Validation, Writing – original draft. GR: Writing – review & editing. ESi: Data curation, Formal analysis, Validation, Writing – original draft. ESk: Data curation, Validation, Writing – original draft. LB: Writing – review & editing. PV: Supervision, Writing – review & editing. GG: Writing – review & editing. ND: Supervision, Writing – review & editing.
